# The effect of experimental pollinator decline on pollinator‐mediated selection on floral traits

**DOI:** 10.1002/ece3.10706

**Published:** 2023-11-09

**Authors:** Kaitlyn S. Brown, Christina M. Caruso

**Affiliations:** ^1^ Department of Integrative Biology University of Guelph Guelph Ontario Canada

**Keywords:** floral evolution, floral trait, phenotypic selection, pollen limitation, pollination, pollinator decline, pollinator‐mediated selection

## Abstract

Human‐mediated environmental change, by reducing mean fitness, is hypothesized to strengthen selection on traits that mediate interactions among species. For example, human‐mediated declines in pollinator populations are hypothesized to reduce mean seed production by increasing the magnitude of pollen limitation and thus strengthen pollinator‐mediated selection on floral traits that increase pollinator attraction or pollen transfer efficiency. To test this hypothesis, we measured two female fitness components and six floral traits of *Lobelia siphilitica* plants exposed to supplemental hand‐pollination, ambient open‐pollination, or reduced open‐pollination treatments. The reduced treatment simulated pollinator decline, while the supplemental treatment was used to estimate pollen limitation and pollinator‐mediated selection. We found that plants in the reduced pollination treatment were significantly pollen limited, resulting in pollinator‐mediated selection for taller inflorescences and more vibrant petals, both traits that could increase pollinator attraction. This contrasts with plants in the ambient pollination treatment, where reproduction was not pollen limited and there was not significant pollinator‐mediated selection on any floral trait. Our results support the hypothesis that human‐mediated environmental change can strengthen selection on traits of interacting species and suggest that these traits have the potential to evolve in response to changing environments.

## Graphical abstract

How will native plant populations respond to declines in pollinator populations? In the bee‐pollinated wildflower *Lobelia siphilitica*, pollinator decline intensifies natural selection for taller inflorescences and more vibrant petals, both traits that can make plants more attractive to pollinators. Photo credit: Gavin Hossack.

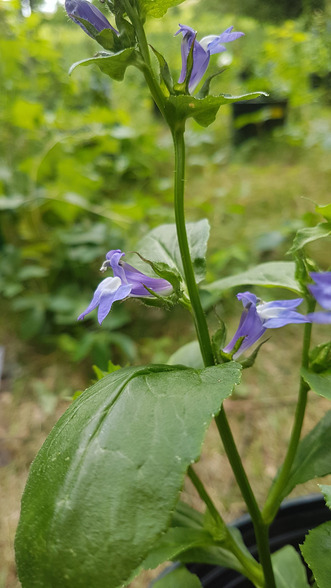

## 1 INTRODUCTION

All species are embedded in a web of interactions with other species, including mutualists, antagonists, and competitors. These species interactions are being altered by human‐mediated changes in the environment, including the intensification of agriculture, the introduction of invasive species, and the shift in climate regimes across the globe (reviewed by Blois et al., 2013; Tylianakis et al., 2008). Such human‐mediated environmental change, by reducing mean fitness, is predicted to strengthen selection on traits that mediate interactions among species (Benkman, 2013; Caruso et al., 2017). However, a recent meta‐analysis found that selection is slightly weaker in human‐disturbed versus natural environments (Fugère & Hendry, 2018), suggesting that human‐mediated environmental change might not consistently reduce mean fitness and strengthen selection on traits that mediate interactions between species (e.g. Campbell & Powers, 2015). Understanding the effect of human‐mediated environmental change on the strength of selection is important because stronger selection should increase the rate of evolution in response to environmental change and thus the likelihood of population persistence (Chevin et al., 2010).

One type of species interaction that has been altered by human‐mediated environmental change is the mutualism between plants and their animal pollinators (Ollerton, 2017). Human‐mediated environmental change has been linked to declines in pollinator populations (González‐Varo et al., 2013), which can reduce the number and quality of pollinator visits to plants (Burkle et al., 2013). This reduction in pollination services, by increasing pollen limitation of reproduction, can reduce female fitness (i.e. seed production; Rodger et al., 2021). Seed production is particularly likely to decrease in species that cannot autonomously self‐pollinate and thus are dependent on pollinators to produce seeds (Bennett et al., 2020). If mean seed production is reduced, then the opportunity for selection should increase (Rundle & Vamosi, 1996), resulting in stronger pollinator‐mediated selection on floral traits that increase pollinator attraction or pollen transfer efficiency as pollinators decline (Benkman, 2013; Caruso et al., 2017; Sletvold & Ågren, 2016). Such strong pollinator‐mediated selection on floral traits would suggest that plants have the potential to rapidly evolve in response to pollinator decline (reviewed in Thomann et al., 2013).

The hypothesis that pollinator decline, by increasing the magnitude of pollen limitation, strengthens pollinator‐mediated selection on floral traits has received mixed support. Experimentally simulating pollinator decline by excluding pollinators for a portion of the flowering span strengthened selection on floral traits in some study systems (e.g. *Impatiens capensis*; Panique & Caruso, 2020), but not in others (e.g. *Passiflora incarnata*; Dai & Galloway, 2013), resulting in a relatively small effect of pollinator exclusion on selection across study systems in a recent meta‐analysis (Caruso et al., 2019). However, previous studies that experimentally simulated pollinator decline (with the exception of Sletvold & Ågren, 2016) had two limitations. First, previous studies did not use a supplemental hand‐pollination treatment to estimate pollen limitation (Bennett et al., 2018), and thus did not test the prediction that pollinator decline increases the magnitude of pollen limitation. Second, previous studies did not use a supplemental hand‐pollination treatment to measure pollinator‐mediated selection on floral traits (Sletvold & Ågren, 2010), and thus did not directly test the prediction that pollinator decline strengthens pollinator‐mediated selection on floral traits. Instead, previous studies measured net (i.e. pollinator‐mediated + non‐pollinator‐mediated) selection, which can differ in strength and direction from pollinator‐mediated selection on floral traits (e.g. Benoit & Caruso, 2021). Consequently, more studies are needed to determine whether pollinator decline commonly strengthens pollinator‐mediated selection on floral traits.

We tested the hypothesis that pollinator decline, by increasing the magnitude of pollen limitation, strengthens pollinator‐mediated selection on floral traits of the wildflower *Lobelia siphilitica*. We studied *L. siphilitica* for two reasons. First, because *L*. *siphilitica*'s primary pollinators (*Bombus* spp.; Flanagan et al., 2011) have been declining in portions of its range (e.g. Ontario [Colla & Packer, 2008]), it is a relevant system for studying the effect of pollinator decline on selection on floral traits. Second, because *L. siphilitica* has a specialized form of secondary pollen presentation (i.e. the pump mechanism; Eisen et al., 2017) that prevents autonomous self‐pollination (Johnston, 1992), it is the type of pollinator‐dependent species that should be particularly vulnerable to increased pollen limitation as pollinators decline (Bennett et al., 2020). In a previous study of *L. siphilitica* (Hossack & Caruso, 2023), we found that experimentally simulating pollinator decline strengthened net selection on inflorescence height, a trait that can increase pollinator attraction. However, we did not use a supplemental hand‐pollination treatment to test whether pollinator decline strengthens pollinator‐mediated selection on floral traits of *L. siphilitica* by increasing the magnitude of pollen limitation.

To test whether pollinator decline, by increasing the magnitude of pollen limitation, strengthens pollinator‐mediated selection on floral traits, we measured two female fitness components and six floral traits of *L. siphilitica* plants exposed to supplemental hand‐pollination, ambient open‐pollination, or reduced open‐pollination treatments. The reduced treatment simulated pollinator decline (as in Hossack & Caruso, 2023), while the supplemental treatment was used to estimate pollen limitation (Bennett et al., 2018) and pollinator‐mediated selection (Sletvold & Ågren, 2010). These estimates of pollen limitation and pollinator‐mediated selection were used to answer the following questions:

1. Does experimentally reducing pollination to simulate pollinator decline increase the magnitude of pollen limitation?
2. Does experimentally reducing pollination to simulate pollinator decline strengthen pollinator‐mediated selection on floral traits?

## 2 MATERIALS AND METHODS

### 2.1 Study species

*Lobelia siphilitica* (Campanulaceae) is an herbaceous, short‐lived perennial wildflower that is native to wetlands and woodlands of eastern North America (Johnston, 1991). *Lobelia siphilitica* plants produce either hermaphroditic or female flowers (i.e. gynodioecy; Caruso & Case, 2007) from late July to September (Parachnowitsch & Caruso, 2008). Flowers have blue to purple petals (Caruso et al., 2010) and are produced along a central raceme (Johnston, 1991), although additional lateral racemes can also be produced (Parachnowitsch & Caruso, 2008). Each flower on a hermaphroditic plant is initially in the male phase and enters the female phase only after all pollen has been dispensed (i.e. complete dichogamy; Dudle, 1999).

### 2.2 Germination and growth

The plants for our experiment were grown from seeds collected from 49 open‐pollinated *L. siphilitica* plants (hereafter maternal families) in the T.J. Dolan Natural Area (Stratford, ON, Canada; 43.2206° N, 81.0035° W). Seeds from these maternal families were either exposed to cold stratification for eight weeks (Johnston, 1992) or treated with a solution of distilled water, bleach, and 70% ethanol (Dudle et al., 2001) to break dormancy. For each dormancy‐breaking method, 90 seeds per maternal family were sown into plug trays filled with Sunshine Mix (Sun Gro Horticulture Canada Ltd., Vancouver, BC, Canada), placed in standing water, and haphazardly arranged along a greenhouse bench in the University of Guelph Phytotron (Guelph, ON, Canada). Seedlings (*N* = 1–50) from 37 maternal families with adequate germination were transplanted into 635 mL Deepots (Stuewe and Sons Inc., Tangent, OR, USA) and randomly assigned a position along the greenhouse bench. Plants were fertilized with eight pellets of Nutricote total 13–13–13 with micronutrients (Plant Products, Brampton, ON, Canada), and watered as necessary. To ensure that all plants would flower before the end of the experiment, the 400 largest plants were selected for inclusion in the experiment. The remaining 80 plants served as pollen donors for the supplemental hand‐pollination treatment (see Experimental Design, below). Prior to being moved into the field, plants were transplanted into 8.7 L pots. All plants included in the experiment were hermaphroditic because the population that we collected seeds from contained very few females (~1%; Caruso & Case, 2013).

### 2.3 Experimental design

Of the 400 *L*. *siphilitica* plants that we transplanted, 100 were randomly assigned to each of four field sites at the Koffler Scientific Reserve at Jokers Hill (King City, ON, Canada; 44.02976° N, 79.533463° W). The field sites were fenced to exclude deer and separated by at least 200 m in old fields that contained perennial grasses such as *Bromus*. The field site did not contain naturally occurring *L. siphilitica*, but did contain co‐flowering species such as *Daucus carota*, *Asclepias syriaca*, and *Vicia cracca*. Within each field site, *L*. *siphilitica* plants were separated by 0.5 m, and the surrounding vegetation was trimmed to a maximum height of ~20 cm to ensure that it would not exceed the top of the pots.

Within each field site, preflowering *L*. *siphilitica* plants were randomly assigned to one of two open‐pollination treatments: ambient open pollination (*N* = 50 per field site) or reduced open pollination (*N* = 25 per field site). The treatments differed in sample size because our experiment was part of a larger study of the causes of selection on floral traits (Benoit & Caruso, 2021). Plants in the ambient treatment were not manipulated. Plants in the reduced treatment were covered in bridal veil netting for 2 days, then left uncovered for 2 days, alternating every 2 days for the duration of the experiment (i.e. a ~50% reduction in pollinator access; Hossack & Caruso, 2023).

In addition to the open‐pollination treatments, we also randomly assigned *N* = 25 preflowering *L*. *siphilitica* plants per field site to the supplemental hand‐pollination treatment. Fewer plants were assigned to the supplemental treatment than the ambient open‐pollination treatment because fitness is expected to be less variable in the supplemental treatment (Sletvold & Ågren, 2014). Plants in the supplemental treatment were hand pollinated every two days for the duration of the experiment (as in Rivkin et al., 2015). Specifically, all female‐phase flowers on plants in the supplemental treatment received pollen from a haphazard sample of at least 5 of the 80 pollen donors. Because supplemental hand‐pollination ensures that all plants (regardless of their floral traits) receive abundant pollen to fertilize their ovules, it can be used to estimate both pollen limitation as the difference in seed and fruit production between the hand‐ and open‐pollination treatments (Bennett et al., 2018) and pollinator‐mediated selection on floral traits as the difference in selection between hand‐ and open‐pollination treatments (Sletvold & Ågren, 2010).

The treatments started on June 18–19, 2015, and continued until August 15–16, 2015, when most plants had finished flowering. Plants were then moved to the University of Guelph Bovey Greenhouse (Guelph, ON, Canada), where they remained until their fruits matured.

### 2.4 Trait and fitness measurements

For all *L*. *siphilitica* plants in the experiment, we measured two traits of inflorescences that could affect pollinator attraction: inflorescence height and daily display size. Inflorescence height was measured as the distance from the soil to the topmost flower at the end of the experiment. Daily display size was measured by counting the number of open flowers on each plant every four days for the duration of the experiment. These counts were then used to calculate the mean number of open flowers per day for each plant.

We also measured two flower color traits that could affect pollinator attraction: petal brightness and petal chroma. To measure brightness and chroma, we collected one of the first five open flowers on each plant (as in Wassink & Caruso, 2013) and measured reflectance spectra using an Ocean Optics USB2000 spectrophotometer (as in Caruso et al., 2010). These spectra were then analyzed using principal components analysis (Grill & Rush, 2000). The first principal component (PC1) estimated petal brightness, the amount of light reflected at all wavelengths; *L. siphilitica* flowers with higher values for PC1 were lighter in color, whereas flowers with lower values for PC1 were darker in color. The second principal component (PC2) estimated petal chroma, the amount of gray mixed in with the primary color; *L. siphilitica* flowers with higher values for PC2 were vibrant in color, whereas flowers with lower values for PC2 were faded in color. Although *L*. *siphilitica* flowers can vary in hue from blue to purple (Caruso et al., 2010), all plants in our population produced purple flowers and thus petal hue was not measured.

In addition to flower color, we measured two floral morphology traits, one that could affect pollinator attraction (corolla projected area) and one that could affect pollen transfer efficiency (corolla tube length). To measure these traits, the same flowers used to measure petal color were photographed using a flatbed scanner and WinRhizo (Regents Instrument Inc., Sainte‐Foy, QC, Canada). The images were then measured using ImageJ (Abràmoff et al., 2004). Corolla projected area was measured as the area of the three fused lower petal lobes (i.e. the lower lip) and two fused upper petal lobes (i.e. the upper lip; Zung et al., 2015). Corolla tube length was measured as the distance from the base of the flower to the intersection of the upper and lower lips.

We estimated two female fitness components, both of which we have previously used to measure selection on floral traits of *L. siphilitica* (e.g. Caruso et al., 2003; Caruso & Yakobowski, 2008; Parachnowitsch & Caruso, 2008): fruits per plant and seeds per plant. To estimate fruits per plant, we counted the number of fruits produced by each plant at the end of the experiment. To estimate seeds per plant, we multiplied the number of fruits per plant times the mean seeds per fruit. Seeds per fruit was estimated for *N* (1 SE) = 15.2 (0.19) randomly sampled fruits per plant. These fruits were sampled by marking the top‐ and bottom‐most open flower on the raceme every ~10th day, and randomly sampling 3 fruits from each 10‐day section. Three fruits were also randomly sampled from any lateral racemes. The seeds in each fruit were weighed, and the mass of seeds in each fruit was divided by the mean mass of 50 seeds (estimated from a random sample of *N* = 62 fruit) and then multiplied by 50 (as in Johnston, 1991) to estimate the number of seeds per fruit.

### 2.5 Statistical analysis

To test whether experimentally reducing pollination to simulate pollinator decline can increase the magnitude of pollen limitation, we quantified pollen limitation as the log response ratio $\ln\left(\frac{\omega_{\text{hand}}}{\omega_{\text{open}}}\right)$ (Bennett et al., 2018), where $\omega_{\text{hand}}$ is the mean fitness component in the supplemental hand‐pollination treatment and $\omega_{\text{open}}$ is the mean fitness component in the ambient or reduced open‐pollination treatment. The log response ratio will be zero when reproduction is not limited by pollen receipt and will increase as the magnitude of pollen limitation increases. We then tested (1) whether reproduction was limited by pollen receipt in the ambient and reduced treatments and (2) whether the magnitude of any pollen limitation was greater in the reduced than in the ambient treatment using one‐tailed planned comparisons. To calculate these planned comparisons, we first fit an ANOVA model that included a fitness component as the dependent variable, treatment as a fixed factor, and field site as a blocking factor. Fitness components were then compared between treatments using one‐tailed planned comparisons. If $\omega_{\text{hand}}>\omega_{\text{ambient}}$, then we concluded that reproduction was limited by pollen receipt in the ambient treatment. If $\omega_{\text{hand}}>\omega_{\text{reduced}}$, then we concluded that reproduction was limited by pollen receipt in the reduced treatment. And if $\omega_{\text{ambient}}>\omega_{\text{reduced}}$, then we concluded that experimentally reducing pollination to simulate pollinator decline increased the magnitude of pollen limitation.

To test whether experimentally simulating pollinator decline can strengthen pollinator‐mediated selection ($\beta_{\text{poll}}$) on floral traits, we estimated $\beta_{\text{poll}}$ in the ambient and reduced open‐pollination treatments. To estimate $\beta_{\text{poll}}$, we first measured standardized directional selection gradients (Lande & Arnold, 1983) in the supplemental $\left(\beta_{\text{supplemental}}\right)$, ambient $\left(\beta_{\text{ambient}}\right)$, and reduced $\left(\beta_{\text{reduced}}\right)$ treatments using general linear models. Each model included a female fitness component as the dependent variable, six floral traits as continuous independent variables, and field site as a blocking factor. Correlations among the six floral traits included in our models were <0.4 and variance inflation factors were <2, indicating that our estimates of selection gradients were not affected by multicollinearity. Floral traits were standardized to a mean of zero and a variance of one (z‐transformation; Sokal & Rohlf, 1995), and fitness was relativized by dividing by mean fitness (Lande & Arnold, 1983). Traits were standardized and fitness was relativized separately within each treatment to facilitate comparisons with other studies; however, selection gradients where fitness was relativized across treatments (sensu terHorst et al., 2015) were similar (data not shown). $\beta_{\text{supplemental}}$ estimates non‐pollinator‐mediated selection (e.g. Trunschke et al., 2017), whereas $\beta_{\text{ambient}}$ estimates net (i.e. pollinator‐mediated + non‐pollinator‐mediated) selection in the ambient pollination environment and $\beta_{\text{reduced}}$ estimates net selection in the reduced pollination environment that simulated pollinator decline. These selection gradients were then used to estimate $\beta_{\text{poll}}$ in the ambient treatment as $\beta_{\text{ambient}}-\beta_{\text{supplemental}}$ and $\beta_{\text{poll}}$ in the reduced treatment as $\beta_{\text{reduced}}-\beta_{\text{supplemental}}$.

To test whether there was significant pollinator‐mediated selection in the ambient and reduced treatments, we used planned comparisons. To calculate these planned comparisons, we first fit a general linear model (GLM) that included a fitness component as the dependent variable; six floral traits as continuous independent variables; field site as a blocking factor; treatment as a categorical independent variable; and all floral trait × treatment terms. We then used the floral trait × treatment terms to estimate planned comparisons of selection in the ambient versus supplemental treatments ($\beta_{\text{ambient}}-\beta_{\text{supplemental}}=0$) and the reduced versus supplemental treatments ($\beta_{\text{reduced}}-\beta_{\text{supplemental}}=0$). If $\beta_{\text{ambient}}-\beta_{\text{supplemental}}\neq 0$, then we concluded that pollinator‐mediated selection was significant in the ambient open‐pollination treatment. If $\beta_{\text{reduced}}-\beta_{\text{supplemental}}\neq 0$, then we concluded that pollinator‐mediated selection was significant in the reduced open‐pollination treatment. If $\beta_{\text{poll}}$ was significant in the reduced treatment, but not in the ambient treatment, then we concluded that pollinator decline strengthens pollinator‐mediated selection on floral traits.

Based on previous pollinator reduction and pollen supplementation experiments (e.g. Caruso et al., 2010; Hossack & Caruso, 2023), we assumed that manipulating pollination would not affect the expression of floral traits in *L*. *siphilitica*. To test this assumption, we compared floral traits between treatments using analysis of variance (ANOVA). The ANOVA model included one of six floral traits as the dependent variable, treatment as a fixed factor, and field site as a blocking factor. When the treatment term in the model was significant, we used Tukey's HSD to test for pairwise differences between treatments. If the treatment term was nonsignificant, then we concluded that manipulating pollination did not affect the expression of floral traits in *L. siphilitica*.

For all models, the assumption of normality was tested by visually examining residuals, and the assumption of equality of variances was tested either by visually examining residuals or by using Levene's test. These assumptions were met for all models except for the ANOVA testing whether petal brightness differed between treatments (Table 4). To determine whether the results from this model were robust, we confirmed that petal brightness did not differ between treatments using a Kruskal–Wallis test (χ^2^ = 3.415; df = 2; *p* = .181). Consequently, we report and interpret the ANOVA for petal brightness (Table 4).

## 3 RESULTS

To test whether experimentally reducing pollination to simulate pollinator decline increases the magnitude of pollen limitation (estimated as the log response ratio), we compared the fruit and seed production of *L. siphilitica* plants in the supplemental, ambient, and reduced pollination treatments (Table 1). We found that the number of fruits (*t*_380_ = −0.111; *p* = .544) and seeds (*t*_336_ = 0.988; *p* = .162) per plant did not differ between the supplemental and ambient treatments, indicating that reproduction was not significantly pollen limited in the ambient treatment (i.e. a log response ratio ≅ 0; Table 1). In contrast, we found that the number of seeds per plant (but not fruits per plant; *t*_380_ = 1.634; *p* = .052) did differ between the supplemental and reduced treatments (*t*_336_ = 3.166; *p* = .001): seed production was ~30% higher in the supplemental treatment than in the reduced treatment, indicating that reproduction was significantly pollen limited in the reduced treatment (i.e. a log response ratio > 0; Table 1). In addition, plants in the reduced treatment produced ~11% fewer fruits and ~18% fewer seeds than plants in the ambient treatment (Table 1). This significant difference in fruit (*t*_380_ = 2.024; *p* = .022) and seed (*t*_336_ = 2.656; *p* = .004) production between the ambient and reduced treatments suggests that experimentally reducing pollination to simulate pollinator decline increased the magnitude of pollen limitation in *L. siphilitica*.

**TABLE 1:** Mean (±1 SE [*N*]) female fitness components for *Lobelia siphilitica* plants exposed to each of three pollination treatments.

	Treatment			Pollen limitation	
	Supplemental hand	Ambient open	Reduced open	Ambient open	Reduced open
Fruits per plant	88.7 ± 3.8 (93)	89.5 ± 2.7 (195)	79.9 ± 3.9 (98)	−0.009 ± 0.052	0.104 ± 0.065
Seeds per plant	56,497 ± 2811 (86)	53,079 ± 2201 (168)	43,603 ± 2286 (88)	0.062 ± 0.065	0.259 ± 0.072

*Note*: Plants in the supplemental treatment were hand pollinated. Plants in the ambient treatment were open pollinated. Plants in the reduced treatment were covered with mesh bags to reduce pollinator access by 50%. Pollen limitation (±1 SE) was estimated as the log response ratio. Standard errors for the log response ratios were calculated following Hedges et al. (1999).

To test whether experimentally reducing pollination to simulate pollinator decline strengthens pollinator‐mediated selection ($\beta_{\text{poll}}$) on floral traits, we compared selection on floral traits of plants in the supplemental, ambient, and reduced pollination treatments (Tables 2 and 3). We found that selection on floral traits did not differ between the supplemental and ambient treatments, indicating that there was not significant pollinator‐mediated selection on any floral trait in the ambient treatment (i.e. $\beta_{\text{poll}}≃0$; Table 3). In contrast, we found that selection on two traits (petal chroma and inflorescence height) did differ between the supplemental and reduced treatments (i.e. $\beta_{\text{poll}}\neq 0$), indicating that there was significant pollinator‐mediated selection on these traits in the reduced treatment (Table 3). Specifically, pollinators in the reduced treatment exerted selection for taller inflorescences via fruits/plant and selection for more vibrant petals (i.e. higher chroma) via seeds/plant (Table 3). This significant pollinator‐mediated selection in the reduced pollination treatment, but not in the ambient pollination treatment, suggests that experimentally reducing pollination to simulate pollinator decline strengthened pollinator‐mediated selection on floral traits.

**TABLE 2:** Standardized directional selection gradients ±1 SE (*p*‐value) via two female fitness components on six floral traits of *Lobelia siphilitica* exposed to each of three pollination treatments.

Fitness component	Trait	Treatment		
		Supplemental hand $\left(\beta_{\text{supplemental}}\right)$	Ambient open $\left(\beta_{\text{ambient}}\right)$	Reduced open $\left(\beta_{\text{reduced}}\right)$
Fruits per plant	Inflorescence height (cm)	0.006 ± 0.041 (0.889)	0.039 ± 0.028 (0.166)	0.159 ± 0.054 (0.004)
	Daily display size	0.241 ± 0.039 (2.78 × 10^−8^)	0.201 ± 0.028 (9.42 × 10^−12^)	0.209 ± 0.049 (5.51 × 10^−5^)
	Petal brightness	0.003 ± 0.038 (0.927)	−0.030 ± 0.027 (0.258)	0.011 ± 0.042 (0.799)
	Petal chroma	−0.123 ± 0.036 (0.001)	−0.080 ± 0.026 (0.003)	−0.084 ± 0.045 (0.063)
	Corolla projected area (cm^2^)	0.095 ± 0.038 (0.014)	0.030 ± 0.028 (0.294)	0.049 ± 0.050 (0.328)
	Corolla tube length (cm)	−0.113 ± 0.042 (0.008)	−0.036 ± 0.028 (0.191)	−0.157 ± 0.050 (0.003)
	Field site	*F*_3,83_ = 1.424 *p* = .242	*F*_3,185_ = 3.713 *p* = .013	*F*_3,88_ = 2.325 *p* = .080
	*N*	93	195	98
Seeds per plant	Inflorescence height (cm)	0.019 ± 0.051 (0.711)	0.083 ± 0.038 (0.033)	0.140 ± 0.060 (0.022)
	Daily display size	0.231 ± 0.048 (6.61 × 10^−6^)	0.219 ± 0.038 (5.09 × 10^−8^)	0.196 ± 0.054 (0.001)
	Petal brightness	−0.041 ± 0.045 (0.361)	−0.073 ± 0.037 (0.048)	−0.018 ± 0.045 (0.694)
	Petal chroma	−0.151 ± 0.043 (0.001)	−0.042 ± 0.036 (0.254)	0.019 ± 0.049 (0.705)
	Corolla projected area (cm^2^)	0.123 ± 0.044 (0.007)	0.057 ± 0.039 (0.144)	0.054 ± 0.056 (0.335)
	Corolla tube length (cm)	−0.101 ± 0.049 (0.043)	−0.015 ± 0.038 (0.693)	−0.031 ± 0.058 (0.599)
	Field site	*F*_3,76_ = 0.040 *p* = .989	*F*_3,158_ = 5.960 *p* = .001	*F*_3,78_ = 0.391 *p* = .760
	*N*	86	168	88

*Note*: Plants in the supplemental treatment were hand pollinated. Plants in the ambient treatment were open pollinated. Plants in the reduced treatment were covered with mesh bags to reduce pollinator access by 50%. *p*‐values indicate whether a selection gradient (estimated from a general linear model) was significantly different from zero. We did not correct the *p*‐Values associated with selection gradients for multiple tests because doing so would increase the likelihood that we will falsely conclude that a trait is not a target of selection as pollinators decline (i.e. an increased Type II error rate). Field site was included as a blocking factor.

**TABLE 3:** Pollinator‐mediated selection ($\beta_{\text{poll}}$ ± 1 SE [*p*‐value]) via two female fitness components on six floral traits of *Lobelia siphilitica* exposed to ambient and reduced open‐pollination treatments.

Fitness component	Trait	Ambient open	Reduced open
Fruits per plant	Inflorescence height (cm)	0.033 ± 0.050 (0.495)	0.153 ± 0.068 (0.015)
	Daily display size	−0.040 ± 0.048 (0.459)	−0.032 ± 0.063 (0.543)
	Petal brightness	−0.033 ± 0.047 (0.699)	0.008 ± 0.057 (0.727)
	Petal chroma	0.043 ± 0.044 (0.546)	0.039 ± 0.058 (0.469)
	Corolla projected area (cm^2^)	−0.065 ± 0.047 (0.298)	−0.046 ± 0.063 (0.370)
	Corolla tube length (cm)	0.077 ± 0.050 (0.229)	−0.044 ± 0.065 (0.381)
Seeds per plant‐	Inflorescence height (cm)	0.064 ± 0.064 (0.328)	0.121 ± 0.079 (0.114)
	Daily display size	−0.012 ± 0.061 (0.945)	−0.035 ± 0.072 (0.703)
	Petal brightness	−0.032 ± 0.058 (0.887)	0.023 ± 0.064 (0.581)
	Petal chroma	0.109 ± 0.056 (0.114)	0.170 ± 0.065 (0.018)
	Corolla projected area (cm^2^)	−0.066 ± 0.059 (0.441)	−0.069 ± 0.071 (0.234)
	Corolla tube length (cm)	0.086 ± 0.062 (0.283)	0.070 ± 0.076 (0.480)

*Note*: Plants in the ambient treatment were open pollinated. Plants in the reduced treatment were covered with mesh bags to reduce pollinator access by 50%. $\beta_{\text{poll}}$ was estimated using standardized directional selection gradients and their SEs (estimated from a general linear model) for plants exposed to ambient, reduced, and supplemental hand‐pollination treatments (Table 2). Specifically, $\beta_{\text{poll}}$ was estimated as $\beta_{\text{ambient}}-\beta_{\text{supplemental}}$ in the ambient treatment and $\beta_{\text{reduced}}-\beta_{\text{supplemental}}$ in the reduced treatment. Standard errors for $\beta_{\text{poll}}$ were estimated as $\sqrt{{\mathrm{SE}}_{\text{supplemental}}^{2}+{\mathrm{SE}}_{\text{ambient}}^{2}}$ for the ambient treatment and $\sqrt{{\mathrm{SE}}_{\text{supplemental}}^{2}+{\mathrm{SE}}_{\text{reduced}}^{2}}$ for the reduced treatment (Chapurlat et al., 2019). *p*‐values next to an estimate of $\beta_{\text{poll}}$ indicate whether there was significant pollinator‐mediated selection in the ambient or reduced open‐pollination treatment (i.e. $\beta_{\text{ambient}}-\beta_{\text{supplemental}}\neq 0$ or $\beta_{\text{reduced}}-\beta_{\text{supplemental}}\neq 0$ in a planned comparison; df = 362 for fruits per plant; df = 318 for seeds per plant).

To test the assumption that manipulating pollination does not affect the expression of floral traits, we compared six traits of *L. siphilitica* between pollination treatments (Table 4). Five of the floral traits did not differ significantly between pollination treatments (Table 4). One trait, corolla tube length, did differ between pollination treatments: corolla tubes were significantly longer for plants in the reduced treatment than for plants in the supplemental treatment (Table 4; Tukey's HSD; *p* = .014). However, corolla tubes of flowers in the reduced treatment were only ~3% longer than tubes of flowers in the supplemental treatment, indicating that any effect of manipulating pollination on the expression of *L. siphilitica*'s floral traits was small in magnitude.

**TABLE 4:** Mean (±1 SE) for six floral traits of *Lobelia siphilitica* plants exposed to each of three pollination treatments.

Trait	Treatment			
	Supplemental hand	Ambient open	Reduced open	*F*_treatment_ (*p*‐value)
Inflorescence height (cm)	45.3 ± 0.90	46.7 ± 0.71	45.4 ± 0.86	1.11 (.331)
Daily display size	16.0 ± 0.74	16.9 ± 0.48	18.2 ± 0.74	2.43 (.090)
Petal brightness	−0.12 ± 0.09	−0.01 ± 0.07	0.15 ± 0.12	1.85 (.158)
Petal chroma	−0.06 ± 0.11	0.05 ± 0.07	−0.10 ± 0.10	1.02 (.362)
Corolla projected area (cm^2^)	1.50 ± 0.04	1.52 ± 0.03	1.61 ± 0.03	2.58 (.077)
Corolla tube length (cm)	1.31 ± 0.01	1.34 ± 0.01	1.35 ± 0.01	4.10 (.018)
*N*	93	195	98	

*Note*: Plants in the supplemental treatment were hand pollinated. Plants in the ambient treatment were open pollinated. Plants in the reduced treatment were covered with mesh bags to reduce pollinator access by 50%. *F*‐ and *p*‐values indicate whether a trait differs between treatments in an ANOVA. df = 2, 380.

## 4 DISCUSSION

To test whether pollinator decline, by increasing the magnitude of pollen limitation, strengthens pollinator‐mediated selection on floral traits, we simulated pollinator decline by experimentally reducing pollinator access to *L. siphilitica* plants. We found that plants in the reduced pollination treatment were significantly pollen limited (Table 1), resulting in pollinator‐mediated selection for taller inflorescences and more vibrant petals (Table 3), both traits that could increase pollinator attraction. This contrasts with plants in the ambient pollination treatment, where reproduction was not pollen limited (Table 1) and there was not significant pollinator‐mediated selection on any floral trait (Table 3). Overall, these results support the hypothesis that pollinator decline, by increasing the magnitude of pollen limitation, strengthens pollinator‐mediated selection on floral traits.

Only one other study (Sletvold & Ågren, 2016) has simulated pollinator decline by experimentally reducing pollinator access and measured pollinator‐mediated (rather than net) selection on floral traits. Consistent with our study, Sletvold and Ågren (2016) found that experimentally reducing pollination strengthened pollinator‐mediated selection on floral traits that can increase pollinator attraction or pollen transfer efficiency, including longer nectar spurs, larger corollas, and taller inflorescences. These consistent results support the prediction that pollinator decline, as a type of human‐mediated environmental change that reduces mean fitness, can intensify pollinator‐mediated selection on traits that mediate interactions among species (Benkman, 2013; Caruso et al., 2017). However, more studies are needed to determine whether the effect of pollinator decline on selection differs between traits such as inflorescence height that increase pollinator attraction and traits such as nectar spur length that affect pollen transfer efficiency. Specifically, future studies should test the prediction (Opedal, 2021) that pollinator decline is more likely to select for attraction than efficiency traits because pollinator decline increases pollen limitation by reducing visitation rate rather than by affecting the fit between flower and pollinator.

Our finding that pollinator decline strengthened pollinator‐mediated selection on floral traits of *L. siphilitica* (Table 3) by increasing the magnitude of pollen limitation (Table 1) is consistent with the hypothesis that pollen limitation is an important determinant of the strength of pollinator‐mediated selection (Benkman, 2013; Caruso et al., 2017; Sletvold & Ågren, 2016). However, the relationship between pollen limitation and pollinator‐mediated selection is predicted to be nonlinear and accelerating (Benkman, 2013), and thus any increase in pollen limitation may have little effect on selection in populations where pollen limitation is currently modest (Caruso et al., 2019). Contrary to this prediction, we found that pollinator decline can strengthen pollinator‐mediated selection on floral traits of *L. siphilitica*, a species where reproduction of hermaphrodites is often not pollen limited (Caruso et al., 2010; Parachnowitsch & Caruso, 2008; Rivkin et al., 2015). Consequently, our results suggest that pollinator decline could strengthen pollinator‐mediated selection on floral traits in populations that vary greatly in the magnitude of pollen limitation. But pollen limitation is not the only determinant of the strength of pollinator‐mediated selection (Bartkowska & Johnston, 2015; Sletvold & Ågren, 2014), and future studies should test whether pollinator decline can also affect selection by changing the functional relationship between floral traits and pollinator attraction.

We found that experimentally reducing pollinator access to simulate pollinator decline caused stronger pollinator‐mediated selection for taller inflorescences in *L. siphilitica* (Table 3). This result is consistent with our previous study of the effect of simulated pollinator decline on net (rather than pollinator mediated) selection on floral traits of *L. siphilitica* (Hossack & Caruso, 2023), which also found stronger selection for taller inflorescences in hermaphroditic plants. Such a consistent effect of simulated pollinator decline on selection on inflorescence height of *L. siphilitica* is particularly striking because the current study and Hossack and Caruso (2023) were done in different years (2015 vs. 2020), at different field sites (King City vs. Guelph, ON, Canada), using plants grown from seeds collected from populations with different sex ratios (1% vs. 36% females; Caruso & Case, 2013). We predict that inflorescence height will be a common target of selection as pollinators decline in primarily bumblebee‐pollinated species such as *L. siphilitica* for two reasons. First, taller inflorescences can be more attractive to bumblebee pollinators (e.g. Donnelly et al., 1998; Zu & Schiestl, 2017). For example, plants that evolved in response to artificial selection for taller inflorescences were more likely to be visited by bumblebees than plants that evolved in response to selection for shorter inflorescences, as expected if taller inflorescences attract bumblebees (Zu & Schiestl, 2017). Second, inflorescence height can be a target of bumblebee‐mediated selection (e.g. Galen, 1989; Gervasi & Schiestl, 2017). For example, taller inflorescences evolved in experimental *Brassica rapa* populations pollinated by bumblebees, but not in replicate populations pollinated by hoverflies, as expected if bumblebees select for taller inflorescences (Gervasi & Schiestl, 2017). Overall, this evidence that a taller inflorescence could be a common target of selection as pollinators decline suggests that future studies of the effect of pollinator decline on floral evolution should include inflorescence height as a focal trait.

In addition to selection for taller inflorescences, we also detected pollinator‐mediated selection for more vibrant petals (i.e. higher petal chroma) in the reduced pollination treatment (Table 3). Because previous studies that experimentally reduced pollination did not measure the effect of simulated pollinator decline on selection on flower color traits (e.g. Hossack & Caruso, 2023; Sletvold & Ågren, 2016), we do not know whether these traits will be a common target of selection as pollinators decline. However, our results suggest that any effect of pollinator decline on selection on flower color traits may be difficult to detect because these traits commonly experience non‐pollinator‐mediated selection (e.g. Caruso et al., 2010; Sletvold et al., 2016). Specifically, when we estimated selection via seeds per plant in the reduced pollination treatment, we detected non‐pollinator‐mediated selection for less vibrant petals ($\beta_{\text{supplemental}}$ = −0.151; Table 2) that conflicted with pollinator‐mediated selection for more vibrant petals ($\beta_{\text{poll}}=0.170$; Table 3). This conflict between pollinator‐mediated and non‐pollinator‐mediated selection resulted in no net selection on petal chroma in the reduced pollination treatment ($\beta_{\text{reduced}}$ = 0.019; Table 2). Consequently, if we had not estimated both pollinator‐mediated and non‐pollinator‐mediated selection, we would have erroneously concluded that petal chroma was not a target of selection as pollinators decline in *L. siphilitica*. Overall, the conflict between non‐pollinator‐mediated and pollinator‐mediated selection on petal chroma suggests that evolutionary responses to pollinator decline, like responses to other types of human‐mediated environmental change (reviewed by Lau & terHorst, 2020), should be studied in the context of other biotic and abiotic environmental factors.

Our experiment had two primary limitations. First, we measured selection on traits of greenhouse‐grown plants. Relative to plants growing in situ in natural populations, greenhouse‐grown *L. siphilitica* can produce more flowers and fruits (Parachnowitsch & Caruso, 2008), suggesting that their reproduction is less resource limited. This reduction in resource limitation could have increased our power to detect an effect of experimentally simulated pollinator decline on pollinator‐mediated selection (Sapir, 2017). Second, we tested whether pollinator decline intensified selection on floral traits via female fitness components, but pollinator decline could also affect selection via male fitness components (Thomann et al., 2013). Whether pollinator decline will have concordant or conflicting effects on selection via male vs. female fitness depends both on whether attractive floral traits that increase pollen receipt also commonly increase pollen export (reviewed by Ashman & Morgan, 2004; Delph & Ashman, 2006) and on the magnitude of pollen limitation (Briscoe Runquist et al., 2017). If selection on floral traits via male and female fitness conflict, then floral evolution in response to pollinator decline could be limited.

In conclusion, our results support the hypothesis that pollinator decline, by increasing the magnitude of pollen limitation, strengthens pollinator‐mediated selection on floral traits. For plants in the ambient pollination treatment, we found that reproduction was not pollen limited (Table 1), and there was not significant pollinator‐mediated selection on any floral trait (Table 3). But for plants in the reduced pollination treatment that simulated pollinator decline, we found that reproduction was pollen limited (Table 1), and there was significant pollinator‐mediated selection for taller inflorescences and more vibrant petals (Table 3). Given that the strength of selection is a key determinant of the rate of evolution, our results suggest that plant populations have the potential to rapidly evolve more attractive floral traits (Thomann et al., 2013), which could increase the likelihood of population persistence as pollinators decline (Chevin et al., 2010). However, the interaction between plants and their pollinators is not the only type of species interaction that is being altered by human‐mediated environmental change (reviewed by Lau & terHorst, 2020), and different types of species interactions may have different effects on the strength of selection (Benkman, 2013). Consequently, more studies are needed to predict when selection on traits that mediate interactions among species will and will not be strengthened by human‐mediated environmental change.

## AUTHOR CONTRIBUTIONS

**Kaitlyn S. Brown:** Conceptualization (equal); data curation (equal); formal analysis (equal); investigation (lead); writing – original draft (lead); writing – review and editing (supporting). **Christina M. Caruso:** Conceptualization (equal); data curation (equal); formal analysis (equal); funding acquisition (lead); writing – review and editing (lead).

## FUNDING INFORMATION

This work was supported by a Summer Research Assistantship from the University of Guelph College of Biological Science (awarded to KSB) and a discovery grant from the Natural Science and Engineering Research Council of Canada (#400152, awarded to CMC).

## CONFLICT OF INTEREST STATEMENT

The authors have no competing interests to declare.

## Data Availability

Data and code are archived on Dryad (https://datadryad.org/stash/share/Hgffj6qymEEyIh9e8q1k5tY1Ucd_jjVuVQzytDRDc98).
